# Pharmacogenetic approaches in the treatment of alcohol use disorders: addressing clinical utility and implementation thresholds

**DOI:** 10.1186/1940-0640-9-20

**Published:** 2014-09-13

**Authors:** Christian S Hendershot

**Affiliations:** 1 Campbell Family Mental Health Research Institute, Centre for Addiction and Mental Health, Toronto, Ontario, Canada; 2 Department of Psychiatry, University of Toronto, Toronto, Ontario, Canada

**Keywords:** Evidence-based medicine, Personalized medicine, Pharmacogenomics, Alcohol, Mu opioid receptor, Asn40Asp, rs1799971

## Abstract

Despite advances in characterizing genetic influences on addiction liability and treatment response, clinical applications of these efforts have been slow to evolve. Although challenges to clinical translation remain, stakeholders already face decisions about evidentiary thresholds for the uptake of pharmacogenetic tests in practice. There is optimism about potential pharmacogenetic applications for the treatment of alcohol use disorders, with particular interest in the *OPRM1* A118G polymorphism as a moderator of naltrexone response. Findings from human and animal studies suggest preliminary evidence for the clinical validity of this association; on this basis, arguments for clinical implementation can be made in accordance with existing frameworks for the uptake of genomic applications. However, generating evidence-based guidelines requires evaluating the clinical utility of pharmacogenetic tests. This goal will remain challenging, largely due to minimal data to inform clinical utility estimates. The pace of genomic discovery highlights the need for clinical utility and implementation research to inform future translation efforts. Near-term implementation of promising pharmacogenetic tests can help expedite this goal, generating an evidence base to enable efficient translation as additional gene-drug associations are discovered.

## Introduction

Initial optimism about the prospect of genomic medicine has given way to the realization that clinical applications will be challenging to identify and implement [1,2]. Efforts to predict disease incidence or severity based on common genetic variants have shown limited success [1], raising questions about the utility of genomic applications in assessing risk for common health conditions. However, advances in pharmacogenetics have already led to treatment innovations in tertiary care contexts. The U.S. Food and Drug Administration currently lists pharmacogenetic content in >100 drug labels [3]. Genetic testing is standard for some therapies, and evidence for potentially actionable pharmacogenetic variants is accumulating quickly [4,5].

The clinical translation of pharmacogenetics research also faces numerous challenges [6-10]. One challenge is that the rapid pace of genomic sciences has left little time to apply and evaluate emergent knowledge in clinical contexts [10-12], resulting in a translational research gap [12,13]. This evidence gap is consistent with the observation that funding initiatives place heavy priority on genomic discovery (T1) research, with a comparatively minute focus on translational (T2–T4) research [6,12]. Further complicating translation efforts are numerous challenges inherent to the clinical implementation of pharmacogenetic tests. At minimum, these challenges include establishing the clinical validity and clinical utility of candidate biomarkers; training health care providers in their application; addressing ethical, legal, and social implications of genetic testing; navigating third-party reimbursement issues; and updating bioinformatics resources to accommodate genomic information [7-9,14-16].

A key question concerns the evidentiary thresholds necessary to justify the adoption of promising pharmacogenetic tests in practice [6,7,9,17,18]. Perspectives on this question will differ across disciplines and clinical scenarios, suggesting the need to navigate this issue as it relates to addiction therapeutics. Because most interventions for alcohol use disorders have only moderate efficacy, identifying prognostic markers of treatment response is a topic of significant interest [19-22]. However, existing research has focused predominantly on the identification or replication of gene–drug response associations, with little discussion about preconditions for clinical implementation. This paper provides a brief discussion of considerations for implementation, emphasizing the need for near-term translation research to inform the clinical utility of pharmacogenetic protocols in alcohol treatment contexts. As a case scenario, evidence for the *OPRM1* A118G polymorphism as a moderator of naltrexone response is discussed. However, these considerations will be relevant for other gene–drug response associations under study in the context of alcohol treatments, summaries of which are provided elsewhere [23-25].

### Evidence for genetic moderation of naltrexone response

Naltrexone is an opioid receptor antagonist and front-line therapy for alcohol dependence. A widely studied single nucleotide polymorphism in exon 1 of the μ-opioid receptor gene (*OPRM1* A118G, rs1799971) is generally considered the most promising genetic moderator of alcohol treatment outcomes [21,22]. Although molecular evidence concerning the functional role of A118G is not conclusive [24,26,27], preclinical and human research implies functional relevance of *OPRM1* for phenotypes related to pain and analgesia, stress response, and response to psychoactive drugs [26]. Of particular clinical relevance are findings that alcohol-dependent participants with the minor (118G) allele show relatively better clinical outcomes (e.g., lower rates of relapse to heavy drinking) during treatment with naltrexone, but not placebo, compared to those homozygous for the 118A variant (for review see [19,24,26,27]). In weighing this evidence, it is important to note instances of nonreplication [28] and the lack of large-scale prospective trials [29]. Nonetheless, the first meta-analysis on this topic [30] reached conclusions consistent with initial promising findings reported by Oslin and others [31,32].

Human laboratory evidence also suggested that *OPRM1* A118G moderated naltrexone-related reductions in the hedonic effects of alcohol [33]. This finding is notable in that stimulant effects of alcohol relate to the risk for heavy drinking [34] and reflect a potential target of naltrexone. The identification of a functional equivalent to *OPRM1* A118G in rhesus monkeys (C77G) has also allowed investigations in primate models [35-37]. Primates with the 77G variant (which is considered analogous to 118G) showed higher levels of alcohol preference and self-administration, and naltrexone preferentially reduced alcohol self-administration in these animals [35,37], providing evidence for cross-species convergence of this finding [19,35]. Therefore, while the association of *OPRM1* with alcohol use disorder etiology remains controversial [26], findings suggest the relevance of *OPRM1* for a phenotype of clinical interest: reduction in alcohol consumption during treatment with naltrexone versus placebo [19,35]. Overall, these findings offer preliminary evidence for the *clinical validity* of *OPRM1* A118G as a marker of therapeutic response.

### Defining sufficient conditions for clinical implementation

A much-debated issue concerns the degree of evidence necessary to incorporate a promising pharmacogenetic test in practice. This topic has received limited public discussion in the alcohol field, despite that decision-making frameworks for determining the uptake of genomic applications are available. For example, the Centers for Disease Control and Prevention (CDC) endorses the ACCE model [38] for evaluating candidate genomic tests for readiness in practice settings. ACCE emphasizes three performance aspects of genetic tests: analytic validity (availability of a reliable laboratory assay for the genetic variant), clinical validity (prognostic value in relation to clinical phenotypes or intermediate phenotypes of interest), and clinical utility (net benefits minus harms of implementing the test in practice). A fourth category addresses ethical, legal and social implications of implementing the genetic test in a specific clinical scenario. The ACCE model includes 44 standard questions to guide evidence-based evaluation, although adaptations have also been made [39]. Example questions are presented in Table 1.

**Table 1:** **Relevant issues for implementation of genetic tests under the ACCE framework**[38]

**ACCE element**	**Primary considerations**	**Example questions**
*Analytic Validity*	Test reliability/precision.	Are test results reliable within and across laboratory settings?
*Clinical Validity*	Genotype-phenotype associations; test sensitivity/specificity and positive/negative predictive value in relation to clinical outcomes; population differences; environmental modifiers.	Do clinically relevant outcomes of pharmacotherapy vary based on genotype in prospective analyses? Do these findings replicate across populations or clinical settings?
*Clinical Utility*	Net cost-benefit profile of testing. Considerations include the usefulness of the test for clinical decisions; any impact of the testing process on patient care; financial costs of testing; economic consequences of health care decisions resulting from testing; facility, personal, and educational requirements associated with testing; informed consent requirements; and clinical risks associated with testing.	What are the net benefits or harms of testing? In which treatment settings are genetic tests feasible or acceptable? Are effective treatment alternatives available under various test result scenarios? How are pharmacogenetic tests best implemented in the clinic? Does genetic testing itself influence patient or provider behaviors?
*Ethical*, *Legal*, *and Social Implications*	Privacy issues; potential for stigmatization; legal and reporting issues; safeguards to protect against legal or ethical infringements.	Could implementation lead to inequities (e.g., racial group disparities in treatment access)? Would test results potentially disclose sensitive information about other health outcomes?

Analytic validity should not pose a concern in the *OPRM1*-naltrexone scenario, since A118G is a single-point mutation and identifiable with commercial assays. As for clinical validity, the evidence for *OPRM1* is by no means conclusive, however, initial findings could be considered promising. Importantly, the ACCE model and similar frameworks do not require conclusive evidence from high-quality randomized trials to evaluate clinical validity. More limited sources of evidence (for example, nonrandomized studies of acceptable quality, systematic reviews of lower-quality studies, or meta-analyses with evidence of heterogeneity) can be considered [5]. In the case of *OPRM1*, the existence of several retrospective trials and subsequent meta-analyses [30,40] is likely sufficient for initial evaluations of clinical validity.

Assuming an encouraging clinical validity profile, implementation decisions will hinge largely on clinical utility, defined broadly as the net effect (benefits minus harms) of implementation [7,41]. Common indices of clinical utility include relevant treatment endpoints (e.g., relapse to heavy drinking), as well as side effects, adverse events, metabolism profiles, or important intermediate phenotypes. Endpoints that can be linked to broader health or economic metrics (e.g., morbidity/mortality, quality-adjusted life-years, treatment costs) are seen as particularly important for determining clinical utility. However, demonstrating that genotype-based treatment protocols improve these outcomes is not necessarily sufficient, because numerous other factors will influence clinical utility in a given context [42]. As one example, a pharmacogenetic protocol that improves clinical outcomes under efficacy scenarios could have zero clinical utility without adequate uptake of the genetic test and/or medication by providers. Similarly, improved treatment endpoints or distal health outcomes do not serve as the only indicators of clinical utility [41]. Proximal or “soft” clinical outcomes can be important in genetic testing scenarios [42]; for instance, personalized treatment protocols could influence treatment motivation, perceived quality of care, and adherence to treatment regimens [42,43]. Also, perceived utility of a genetic test for therapeutic choice could influence uptake of the test or related medications by providers [41,42]. Ethical, psychosocial, and legal implications of genetic testing are also important for informing clinical utility [41,44]. Importantly, numerous parameters of clinical utility exist and most are context-dependent, potentially varying based on the clinical syndrome, population, treatment setting, and other contextual factors [7,39,41].

A major barrier to the clinical translation of pharmacogenetics is the shortfall of data to inform estimates of clinical utility, contributing to the so-called “evidence dilemma” in genomic medicine [6,45]. This gap exists primarily at the T2 stage, such that even the most promising applications are rarely subjected to rigorous research to inform clinical utility profiles and implementation questions [6,12,46]. A consequence of this “T2 bottleneck” is that decisions about clinical implementation must be made absent comprehensive evidence for or against clinical utility—or else deferred indefinitely [6]. With few exceptions [47], very little data is available to inform clinical utility estimates for genetic applications in alcohol treatment settings, although somewhat more progress has been made in the area of nicotine addiction [10,15,48-53]. Given this evidence shortfall, initial clinical recommendations for pharmacogenetic tests must be made without sufficient evidence for or against clinical utility—a scenario common to most of medicine [6,54].

### Addressing implementation in the context of limited evidence

Most genetic tests are not federally regulated, leaving stakeholders (e.g., the scientific community, clinicians, third-party payers) to navigate implementation decisions [42], usually in the context of insufficient evidence [54]. To facilitate evidence-based recommendations, the CDC sponsored the Evaluation of Genomic Applications in Practice and Prevention (EGAPP) initiative. The chief aim of EGAPP is to develop and test guidelines for the systematic evaluation of candidate genomic applications [5]. Candidate applications are subjected to evidence-based review by an expert panel, following the ACCE framework and resulting in published recommendations [55]. The CDC endorses a three-tier evidentiary framework for assessing candidate genomic applications [54,56]. Tier 1 denotes applications with sufficient evidence to recommend clinical use (e.g., *BRCA* testing in the context of family risk for breast and ovarian cancer). Evidence sufficient to discourage clinical use of a test results in assignment to Tier 3. Tier 2 is used for applications with promising evidence for clinical validity, but insufficient evidence to recommend for or against clinical adoption—often reflecting limited data on clinical utility. The vast majority of pharmacogenetic applications will fall under Tier 2 [54]. Other Tier 2 examples include genetic risk indicators routinely assessed in practice, but without clear evidence for clinical utility (e.g., assessing family history during depression screening in primary care). The Clinical Pharmacogenetic Implementation Consortium (CPIC) and other expert groups have endorsed similar three-tier models for evaluating genomic applications [4,57].

The three-tier scheme is noteworthy in that it accommodates clinical implementation in the absence of sufficient evidence for or against clinical utility [54,57]. To aid these decisions, Tier 2 biomarkers can be further triaged based on anticipated risk-benefit profiles. For example, Tier 2 tests with unfavorable risk-benefit profiles can be subcategorized with a recommendation of “do not use,” whereas those with neutral or marginally favorable profiles may receive recommendations such as “consider use in clinical practice” or “use with evidence-based development” [54,57]. Importantly, the latter designations can justify clinical implementation in the absence of comprehensive data on clinical utility. No evidence-based guidelines for gene–drug combinations specific to addiction therapeutics have been published by EGAPP or CPIC. In the absence of formal evidence-based reviews—which will emerge slowly, given an enormous number of potential gene–drug associations—stakeholders will largely be tasked with making initial implementation decisions. Clinical annotations for candidate pharmacogenetic tests [58] and models for evidence-based evaluation of genomic applications [59] are increasingly available to guide these decisions.

### Noninferiority as a basis for implementation

Placebo-controlled, prospective randomized controlled trials (RCTs) will remain the gold standard for validating pharmacogenetic applications. These designs reduce sources of bias and are necessary for testing genotype-based treatment algorithms [19,29]. However, predicating clinical adoption on prospective RCTs alone will mean a protracted path from discovery to translation [12,60]. An alternative view is that noninferiority of pharmacogenetic protocols can be sufficient for initial implementation [60]. From this perspective, tests for which the anticipated risk-benefit profile is neutral or marginally positive could be implemented and evaluated in practice settings prior to establishing clear utility on the basis of RCTs [60]. A noninferiority approach is not a universal solution [61] but can serve several purposes, such as acclimating health care providers and patients to genetic testing; evaluating and refining treatment algorithms; optimizing the design of bioinformatics resources and electronic medical records; and identifying pragmatic barriers to implementation. These steps can generate important information to inform clinical utility profiles and clinical practice protocols [60]. On the other hand, deferring these steps until supportive data from prospective RCTs accumulates will likely delay clinical translation for those tests that ultimately show acceptable clinical utility [13,60]. As noted above, a reliance on traditional RCTs alone is also insufficient because most trials are not designed to collect comprehensive data on clinical utility.

Because a noninferiority rationale assumes no foreseeable net harms, an important ethical consideration is whether pharmacogenetic protocols could compromise access to best-practice interventions. Ideal scenarios for early implementation might include those where a genetic test informs assignment to one of two empirically supported interventions. For instance, evidence for comparable efficacy of naltrexone and cognitive-behavioral therapy (CBT) for alcohol dependence [62] could justify genotype-based assignment (e.g., assigning naltrexone and CBT as the front-line interventions for 118G carriers and noncarriers, respectively), other considerations being equal. However, contextual factors are important to consider. For instance, the range of available therapeutic options will vary across treatment settings. Assuming noninferiority of a pharmacogenetic protocol and availability of both naltrexone and CBT, a more liberal evidentiary threshold might be used to base treatment selection on *OPRM1*. However, in the hypothetical scenario that naltrexone is the only evidence-based treatment available, the evidentiary bar should arguably be higher before restricting use of naltrexone to 118G carriers.

### Developing an evidence base

Drawing from discussions in other disciplines, several recommendations can be made for near-term efforts to promote translation research in addiction therapeutics. A general goal is to evaluate candidate pharmacogenetic applications with approaches that balance methodological rigor with clinical applicability [8,13,54]. To meet this aim, a comprehensive, portfolio-based approach to translation research has been recommended [8,12]. This approach includes an emphasis on alternative research designs (e.g., feasibility studies, pragmatic clinical trials, adaptive designs, observational studies) that can complement RCTs to generate data on clinical utility and implementation questions [7-9,57]. Early translation efforts can prioritize the most promising genetic markers as prototypes, allowing for clinical implementation to be studied concurrent with (rather than being predicated on) prospective RCTs [12]. One critique of this approach is its apparent circularity; that is, candidate tests require initial implementation in order to generate evidence that can inform broader implementation. In fact, this observation illustrates the evidence dilemma in genomic medicine [6]. A proposed solution is that, rather than dictating a linear progression in the steps from bench to bedside, translation research proceeds as an ongoing, iterative process, including bidirectional knowledge exchange between basic and clinical researchers beginning at an early stage [12].

These considerations support an argument for provisional implementation of promising pharmacogenetic protocols, if and when feasible [60]. Such an approach assumes neutral or marginally favorable risk-benefit profiles for these protocols relative to standard care, as well as supportive local conditions for implementation. Determining the ideal implementation conditions in addiction treatment settings will take additional work, although some minimal conditions can be inferred (e.g., financial and administrative support; acceptability of genetic testing by staff and patients; affiliation with a laboratory that meets necessary regulatory standards; the ability to educate providers on genetic protocols). Implementing candidate genetic tests in the context of naturalistic, observational, pragmatic, or comparative effectiveness trials can produce data on specific implementation questions, complementing findings from RCTs to inform clinical utility [13,54]. Ideally, these efforts can be designed to inform specific translation questions, including those relevant to the ACCE criteria. This strategy reflects the chain-of-evidence approach utilized by EGAPP, such that various sources of data are seen as relevant to informing clinical utility [5,57]. The identification of key “early adopter” sites can help to organize implementation research [8]. Large-scale biobanking and prospective genotyping are commonplace in some hospitals [63,64], offering particularly good venues. To ensure financial support, funding streams for translational (T2–T4) pharmacogenetics research in alcohol treatment contexts are likely critical to these aims.

Preliminary evidence on *OPRM1* and naltrexone response makes this a logical prototype for near-term translation research. At the same time, more research is clearly needed to characterize clinical validity and utility profiles for *OPRM1*. For example, assessments of clinical validity require specific attention to test prognostics (sensitivity, specificity, positive predictive value, and negative predictive value) in relation to the clinical endpoint in question [65]. Consensus is needed around the primary clinical endpoint(s) for such analyses, with particular attention to what constitutes a “positive” or “negative” treatment response. Relapse to heavy drinking has served as a common outcome in retrospective trials [30], but alternative definitions are likely important [31]. Ideally, these outcomes should be defined *a priori* and evaluated in prospective studies [29]. Another question concerns the most appropriate comparator condition for sensitivity/specificity estimates [65]. One consideration is that real-world treatment algorithms will not include placebo, and the lack of a single “gold standard” intervention for alcohol dependence further complicates this question. Importantly, clinical validity estimates for most genetic tests will always be imprecise, due to environmental and contextual moderators specific to a given setting [65].

Regarding clinical utility, a key question is whether genotype-based treatment algorithms offer advantages over standard protocols when implemented in clinical practice scenarios. Research protocols that compare genotype-based treatment assignment to conventional methods are needed to address this question. Adaptive treatment designs [21] could be an important element of such studies. In addition to measuring traditional clinical endpoints, such trials should also evaluate proximal outcomes (e.g., medication adherence, physician attitudes) that are ultimately important for informing clinical utility profiles. As noted, such questions can be addressed using prototype scenarios, even if the overall evidence for clinical validity and utility remains limited [43]. As reviewed above, the use of alternative treatment designs, including noninferiority studies and observational studies in naturalistic settings, can also help generate clinical utility data.

Although ethical issues are potentially less obvious in pharmacogenetic scenarios relative to other areas of genetic testing [60], research on these issues is needed. Example issues include privacy considerations related to biobanking [44] and whether ancillary health risk information could be revealed based on test results [66]. A particularly important issue is the potential for racial or ethnic group disparities in access to pharmacogenetic applications or associated treatments [44,67]. For example, the low prevalence of the 118G allele in people of African descent is one potential explanation for limited efficacy of naltrexone in this population [22]. However, using self-reported race as a basis for treatment decisions can have significant ethical and societal implications, potentially exacerbating disparities in health care access and health outcomes [44].

A key end goal is the development of evidence-based guidelines for candidate pharmacogenetic applications. Published guidelines are seen as critical for promoting awareness of candidate applications, enhancing their uptake [4,9,13], and informing third-party reimbursement [6]. Importantly, the process of guideline development is largely stakeholder-driven [54,59], meaning that those in the alcohol research and treatment communities will be tasked with developing and disseminating these guidelines. In the scenario of *OPRM1*, a decade of research following the initial report [32] has generated multiple studies, leading to initial meta-analyses [30,40]. The publication of upcoming prospective trials (and integration of these results in meta-analyses) would provide an ideal time for an updated data synthesis that includes clinical recommendations. Such a process could follow the format of the EGAPP reviews [55]. Reflecting the general lack of clinical utility data, the foreseeable result of such a review is a conclusion of “insufficient evidence” to recommend using *OPRM1* for clinical purposes. The question then becomes whether to recommend provisional implementation, for example, in specific clinical scenarios or for purposes of evidence development. Such a decision depends on whether the aggregate evidence, even if limited, is deemed “encouraging” or “discouraging” overall. Importantly, setting the implementation threshold either too high or too low can have adverse implications for future translation efforts [6].

### Anticipating barriers

Numerous barriers to the clinical implementation of pharmacogenetic tests have been outlined [9,10,12,15,44,50]. Research is needed to evaluate how these barriers apply in alcohol treatment settings, and to identify barriers unique to these settings. A common critique is the potential for adverse responses to genetic testing, including concerns that genetic testing might undermine behavior change efforts. Importantly, evidence for “genetic fatalism” is limited overall [68]. Genetic tests could also have favorable effects on treatment motivation or adherence [43,69,70], and initial evidence suggests acceptability of genetic tests among those seeking treatment for alcohol use disorders [47]. Efforts should be made to address these concerns empirically, rather than deferring to “genetic exceptionalism” (i.e., treating genetic tests with greater scrutiny than other diagnostic tools) [71]. One example of a clear barrier in the *OPRM1* scenario is the low utilization of naltrexone in both general and specialty settings [72], which ultimately limits clinical utility. Numerous other barriers to implementation can be anticipated, some of which will be evident only as implementation efforts progress [7].

## Conclusion

The likelihood that pharmacogenetic protocols will see common use in alcohol treatment scenarios remains unknown. However, there is optimism about the potential for personalized approaches and consensus on the importance of predicting treatment response [19-22,24,67], giving reason to support near-term clinical translation research. Ultimately, the potential public health benefits of pharmacogenetics research are contingent on efficient translation—in turn requiring greater commitment to clinical utility and implementation research [12,45]. Stakeholders in the field will be responsible for setting translational research and funding priorities, navigating implementation issues, and developing consensus guidelines for candidate applications. A clear goal is to generate much-needed evidence on the clinical utility of such applications in treatment settings, in turn leading to evidence-based guidelines for those applications that show the potential for utility. Near-term evaluation of candidate pharmacogenetic protocols in clinical settings can help expedite this goal, generating an evidence base to enable efficient translation as additional gene–drug response associations are discovered.

## Competing interests

The author declares no competing interests.
